# Dissociative Absorption May Contribute to Internet Gaming Disorder Independent of Childhood Trauma and ADHD Symptoms Among Male University Students

**DOI:** 10.5152/eurasianjmed.2021.21179

**Published:** 2022-10-01

**Authors:** Ali Kandeğer, Ümran Egilmez

**Affiliations:** 1 Department of Psychiatry, Selçuk University Faculty of Medicine, Konya, Turkey; 2 Department of Psychiatry, Niğde Ömer Halisdemir University Training and Research Hospital, Niğde, Turkey

**Keywords:** Absorption, ADHD, childhood trauma, dissociation, internet gaming disorder, somatoform dissociation

## Abstract

**Objective:**

We aimed to investigate the relationship between childhood trauma, dissociative experiences, and internet gaming disorder in male university students with probable attention-deficit hyperactivity disorder determined by both childhood and current attention-deficit hyperactivity disorder symptoms.

**Materials and Methods:**

Volunteers were 376 university students who completed a test battery that included a sociodemographic form as well as the Adult ADHD Severity Rating Scale, Wender Utah Rating Scale, Childhood Trauma Questionnaire, Dissociative Experiences Scale, Somatoform Dissociation Questionnaire, and Internet Gaming Disorder Scale—Short Form. Volunteers were divided into 2 groups as with and without probable attention-deficit hyperactivity disorder D based on both childhood and current attention-deficit hyperactivity disorder symptoms.

**Results:**

Childhood Trauma Questionnaire (*t* = −3.94; *P *< .01), Dissociative Experiences Scale (*t* = −5.97; *P *< .01), Somatoform Dissociation Questionnaire (*t* = −3.80; *P *< .01), and Internet Gaming Disorder Scale—Short Form (*t* = −5.21; *P *< .01) scores were significantly higher in the group with probable attention-deficit hyperactivity disorder than in those without. Two different hierarchical regression analysis models in which internet gaming disorder scores were dependent variables showed that dissociative experiences in first model (β = 0.15, *t* = 2.28, *P *= .023) and dissociative absorption in second model (β = 0.22, *t* = 2.76, *P *= .006) were associated with internet gaming disorder after controlling for childhood trauma and attention-deficit hyperactivity disorder symptoms.

**Conclusion:**

Dissociative absorption may contribute to internet gaming disorder independent of childhood trauma and attention-deficit hyperactivity disorder symptoms, however further studies are needed to investigate this claim.

**Main Points**

- Childhood trauma, both psychoform and somatoform dissociation, and internet gaming disorder scores were higher in the group with probable attention-deficit hyperactivity disorder (ADHD) than in those without.
- Dissociative experiences were associated with internet gaming disorder scores independent of childhood trauma and ADHD symptoms.
- Dissociative absorption was the most associated dissociative subfactor with internet gaming disorder.

## Introduction

Attention-deficit hyperactivity disorder (ADHD) is a neurodevelopmental disorder characterized by attention deficit, hyperactivity, and impulsivity, that is childhood-onset. Although ADHD symptoms tend to decrease in adulthood, a considerable proportion of adults with ADHD remain symptomatic, which negatively affects their academic performance, interpersonal relationships, and social functioning.^1^ In adults, the prevalence of ADHD ranges from 2.5% to 5.2%. Unlike the clinical sample, where female: male ratios as high as 1 : 10 were observed, community samples represented a less extreme sex ratio (female : male risk = 1 : 3) in ADHD prevalence in childhood.^2^ Compared to studies on child and adolescent ADHD, adult studies usually showed a more balanced distribution of ADHD prevalence in males and females. Attention-deficit hyperactivity disorder is a predisposing factor for many psychiatric disorders, including anxiety, mood, eating, trauma-related, and substance-related disorders.^3-5^

Studies investigating the relationship between childhood traumatic events and ADHD have suggested that traumatic experiences in childhood may make trauma victims more vulnerable to aggravation of ADHD symptoms.^6^ It has also been stated that childhood ADHD can be a risk factor for exposure to trauma. Trauma-related symptoms such as motor restlessness, emotional lability, and attention problems can mimic, trigger, or reveal ADHD symptoms. While it has been suggested that dissociative experiences and inattention can overlap in the association between ADHD and traumatic symptoms, there are not enough dissociative experience studies to evaluate this claim in individuals with ADHD.^7^

Internet gaming disorder (IGD) is becoming increasingly common today. If gaming becomes harming a person’s social, job-related, family, school, and psychological functioning, this activity can be defined as pathological.^8^ Generally, “pathological gaming” can be defined as repeated, persistent and excessive participation that cannot be controlled, in spite of gaming-related negative outcomes. Studies have reported prevalence rates for IGD ranging from 0.7% to 11.6%, and the male gender is the most prominent risk factor for IGD.^9,10^

Internet gaming disorder has also been associated with depression, social difficulties, ADHD, and substance abuse.^11^ Moreover, IGD has been linked to multiple comorbidities, such as depression, anxiety, aggression, and obsessive-compulsive symptoms. Attention-deficit hyperactivity disorder is both a major comorbidity and risk factor for IGD. It has been suggested that both disorders share common clinical features and neuronal functional connectivity.^12^ Some of the studies have shown that childhood traumatic experiences and dissociative experiences may contribute to IGD in gamers.^13,14^

While studies on IGD are increasing, it is being investigated why gamers are more prone to dissociative experiences and the role of traumatic experiences at this point. In this study, we aimed to compare childhood trauma, dissociative experiences, and IGD symptoms in male young adults who were divided into 2 groups according to the probability of individuals having ADHD determined by current and childhood ADHD symptoms. In addition, we aimed to investigate the association between dissociative experiences and IGD after controlling for childhood trauma and ADHD symptoms.

## Materials and Methods

This study was conducted in a cross-sectional design and using the online survey method, in Selçuk University Central Campus between June 1, 2020, and September 1, 2020. To carry out the study, permission was provided from the Selçuk University Rectorate and approval from the Local Ethics Committee of Selçuk University Faculty of Medicine (decision number: 2020/130).

The participants completed an online survey including a consent form regarding voluntary participation, the socio-demographic form, Wender Utah Rating Scale (WURS), Adult ADHD Severity Rating Scale (ASRS), Dissociative Experiences Scale (DES), Childhood Trauma Questionnaire (CTQ), Somatoform Dissociation Questionnaire (SDQ), and Internet Gaming Disorder Scale—Short Form (IGDS9-SF).

University students studying at Selçuk University Central Campus were included in the study. When the data collection process was over, 454 university students completed the online survey. Since cognitive abilities and executive function may be affected, participants who reported substance use, regular alcohol use, and any current diagnosis of psychiatric disorders were excluded from the research. Three hundred seventy-six participants’ data were subjected to analysis.

### Measurements Tools

#### Wender Utah Rating Scale

Wender Utah Rating Scale is a 25-item, self-report 5-point Likert type scale that retrospectively questions childhood ADHD symptoms. It consists of 5 subscales: irritability (7 questions), affect (5 questions), academic problems (3 questions), behavioral problems/impulsivity (5 questions), and attention deficit (5 questions). The total score of the scale is acquired by adding the scores from all questions.^15^ The cut-off value was determined as 36 points for childhood ADHD symptoms in this study. The study on the reliability and validity of the Turkish scale was conducted by Öncü et al.^16^

### Adult ADHD Self-Report Scale

It is an 18-question self-report scale that measures ADHD symptoms in adults based upon the criteria of the Diagnostic and Statistical Manual of Mental Disorders: Fourth Edition.^15^ The reliability and validity of the Turkish form have been demonstrated.^17^ Higher scores indicate higher ADHD symptoms. It is suggested that the cut-off score should be determined as 30, and this cutoff score is reported to correspond to sensitivity= 0.75, specificity= 0.79, Kappa= 0.44, positive predictive value= 0.46, and negative predictive value= 0.93.^18^ The ASRS is also used as a 6-item screening tool, shows robust psychometric properties, and displays strong concordance with clinicians’ diagnoses. Adult ADHD Severity Rating Scale 6-item, 5-point Likert type scale ranges from 0 (never) to 4 (very often). On screening items 1-3, each response of 2 or more equated to 1 point; each response of 3 or more on screening items 4-6 scored a point. A total score of 4 or more indicated probable ADHD.^19^

Additionally, it is known that ADHD symptoms should begin in childhood. Therefore, a cross-sectional symptom screening in adulthood is not sufficient for the diagnosis of ADHD according to DSM 5.^20^ Therefore, in this study, we used both the ASRS 6-item, which questions the current ADHD symptoms, and the WURS, which queries the ADHD symptoms in childhood, while determining the probability of ADHD.

### Childhood Trauma Questionnaire

The CTQ is a functional and easy-to-use measurement tool that evaluates early life interpersonal traumas retrospectively and quantitatively. The scale developed by Bernstein et al^21^ consists of 28 questions and 5 subscales as emotional abuse, physical neglect, physical abuse, sexual abuse, and emotional neglect. Validity and reliability of CTQ’s Turkish version have been performed with a Cronbach’s alpha value as 0.93.^22^

### Dissociative Experiences Scale

The DES evaluates a broad variety of types of dissociation including both problematic dissociative experiences such as depersonalization–derealization (the feeling of being an outside observer of one’s life—the feeling of being detached from one’s surroundings), absorption (the total allocation of attention to a certain stimulus while being oblivious to surrounding stimuli) and amnesia (sudden retrograde memory loss, unable to remember important personal information), and normal dissociative experiences (e.g., day-dreaming).^23^ Validity and reliability of Turkish version of DES has been performed and was showed to have great reliability and validity, with a Cronbach’s alpha of α = 0.91.^24^

### Somatoform Dissociation Questionnaire

The SDQ is a self-report scale used to assess somatoform dissociation. It contains 20 items. The scale investigating negative symptoms (analgesia, anesthesia, and motor inhibitions) and positive symptoms (taste change, localized pain, and odor preferences/reluctance) can be scored in the range of 20-100.^25^ Sar et al^26^ conducted the Turkish validity and reliability study.

### Internet Gaming Disorder Scale–Short Form

Internet Gaming Disorder Scale–Short Form is a self-report, 5-point Likert-type scale that evaluates the severity of IGD symptoms, questions 9 IGD criteria (tolerance, preoccupation, withdrawal, loss of control, quit other activities, continuation, cheating, escape, and negative consequences) according to DSM 5. It was developed by Pontes and Griffiths^27^ by applying it to gamers from 58 different countries. It was adapted to Turkish by Evren et al^28^ and was found to be reliable with a Cronbach’s alpha of 0.89.

### Analyses

The data were analyzed using the Statistical Package for the Social Sciences 22 (IBM Corp., Armonk, NY, USA) package program. First, descriptive statistics were calculated. Pearson correlation analyses were then performed to investigate correlations among the scale scores. Volunteers were divided into 2 groups as with and without ADHD probability according to ASRS 6-item screening for current ADHD symptoms and WURS screening for childhood ADHD symptoms. Independent variable *t*-tests were used to compare the 2 groups’ numerical data. Finally, 2 hierarchical multiple regression analyses were performed to evaluate the contribution of dissociative experiences to IGD after controlling for age, ADHD symptoms, and childhood trauma. The risk values were calculated within a 95% CI. The significance threshold was accepted as *P* < .05.

## Results

Participants’ ages ranged from 18 to 57 (mean of 21.83 ± 4.05), and 35.1% (n = 132) reported that they smoked, and 59.8% (n = 225) consumed coffee daily. Independent samples *t*-tests revealed that ASRS (*t* = −13.80, *P *< .01), WURS (*t* = −13.07, *P *< .01), CTQ (*t* = −3.94, *P *< .01), DES (*t* = −5.97, *P *< .01), SDQ (*t* = −4.58, *P *< .01), and IGDS9-SF (*t* = −5.87, *P *< .01) scores were remarkably higher in the group with probable ADHD than in those without. Comparisons of the 2 groups’ demographic data and test scores are presented in Table 1.

As presented in Table 2, Pearson correlation analyses revealed that ASRS, WURS, CTQ, DES, SDQ, and IGDS9-SF scores were significantly positively correlated with one another (*P *< .01).

In the first hierarchical regression analysis to test the relationship between dissociative experiences and internet gaming disorder, IGDS9-SF score was considered the dependent variable. The hierarchical regression model yielded a significant *F* value (*F* (4, 371) = 11.75, *P *< .001). The first step of the hierarchical regression analysis, which included probable ADHD, was statistically significant and participants with probable ADHD (β = 0.29, *t* = 5.87, *P *< .001) were at greater risk of IGD. Childhood trauma was controlled in the second step, revealing no significant effect on IGDS9-SF scores (β = 0.09, *t* = 1.86, *P* = .064). In the third step, psychoform and somatoform dissociation scores were included in the analysis. It was determined that DES score (β = 0.15, *t* = 2.28, *P* = .023), which reflects psychoform dissociation, significantly contributed to variance in IGDS9-SF scores.

After the associations were revealed in the first regression analysis, a second regression analysis including the subscales of CTQ and DES was performed and yielded a significant *F* value (*F* (10, 365) = 5.45, *P *< .001). In the second step, no relationship was found between CTQ subscales and IGD scores. In the third step, it was shown that the amnesia (β = −0.19, *t* = −2.63, *P* = .024) and absorption (β = 0.22, *t* = 2.76, *P* = .006) subscales of DES were significantly associated with IGD. These hierarchical regression analyses findings are presented in Table 3.

## Discussion

Our aim in this study is to examine the relationship between childhood trauma, dissociative experiences, and internet gaming disorder in male university students with probable ADHD. The group with probable ADHD had higher CTQ, DES, SDQ, and IGD scores compared to those without. Hierarchical regression analysis revealed that dissociative experiences are associated with IGD independent of childhood trauma and ADHD probability.

The relationship between ADHD and IGD has been well defined in children, adolescents, and adults.^12,29^ In our study, internet gaming scores were significantly higher in the group with probable ADHD than in those without. Additionally, having probable ADHD in hierarchical regression analysis was a strong predictor for IGD. Moreover, IGD and ADHD have been found to have common brain connections. It has been reported that the concept of hyperfocus, which was previously defined in individuals with ADHD, the mental state with the increased attention that decreases the awareness of the environment and time while engaging with their interests (like a hypnotic spell), also experienced in individuals with IGD during gaming activities.^30,31^ This may be a common phenomenon in both ADHD and IGD, but empirical studies are needed, preferably involving brain connectivity analyses.

It is thought that childhood traumas contribute to IGD and gaming activities help individuals to escape negative cognitions associated with trauma.^13,32^ Although our study revealed that increased childhood trauma scores in the group with probable ADHD compared to those without, regression analyzes did not show a relationship between childhood traumas total and sub-scores with IGD. This may be because childhood trauma predicts IGD by only indirectly increasing dissociative symptoms.

Dissociation is defined as the disruption and/or discontinuity in memory, consciousness, identity, behavior, environment, and body perception that is expected to function synchronously to protect the ego from trauma-related outcomes such as negative cognitions and disturbing emotions. This defense mechanism can affect body perception/representation (somatoform dissociation) as well as executive functions and mentation (psychoform dissociation).^33^ Our study reveals that both psychoform and somatoform dissociation scores as well as childhood trauma scores were higher in the probable ADHD group than in those without. Considering the bidirectional relationship between ADHD and childhood trauma, these results were consistent with the literature.^6,7^

Although there are studies on the relationship between dissociative experiences and IGD in the literature, this study is the first to investigate the association between dissociation and IGD in healthy volunteers after controlling for childhood traumas and ADHD symptoms. The novel finding is that psychoform dissociation (not somatoform) was associated with IGD after controlling for childhood trauma and ADHD probability. Moreover, further analysis showed that in particular dissociative absorption was associated with IGD. Absorption is a tendency to become immersed in a single stimulus, either internal (e.g., a thought or an image) or external (e.g., a movie or a game) while neglecting other stimuli.^34^ This may represent an increased focus (hyperfocus) on certain activities, a facilitating mental process for IGD.

The study also has several limitations, however. Participants were not subjected to psychiatric examination, and psychological parameters were evaluated through self-report scales, which may include bias (e.g., social desirability and recall bias). Additionally, the study was conducted in a single center and was cross-sectional in design, making it difficult to generalize its findings. Longitudinal studies with large sample sizes that use functional brain connectivity analyses and objective neurocognitive tests (e.g., the Stroop Color and Word Test) would provide more comprehensive findings.

This study found that male university students with probable ADHD, determined by both childhood and current ADHD symptoms, had higher childhood trauma, dissociative experiences, and internet gaming disorder scores than those without. The novel finding of our study was that dissociative absorption were associated with IGD after controlling for childhood trauma and ADHD symptoms. To better understand the associations between gaming mental states and dissociative experiences, studies that use objective evaluations such as neurocognitive tests and functional brain connectivity analyses are needed.

## Figures and Tables

**Table 1.** Comparison of Demographic Data and Means ± Standard Deviations of Measurement Tools Between Those With and Without Probable ADHD^*^

Present (N = 66)		Absent (N = 310)	
	Probable ADHD				*t*	*P*
	Age (years)	22.14	3.74	21.76			4.11	−0.68	.50
Adult ADHD Self-Report Scale	44.74	7.10	28.10	9.23	−16.34	**<.001**
Wender Utah Rating Scale	51.20	11.03	26.87	14.24	−15.40	**<.001**
Childhood Trauma Questionnaire	44.27	12.13	38.19	11.22	−3.94	**<.001**
*Emotional Neglect*	14.15	4.60	12.30	4.89	−2.82	**.005**
*Emotional Abuse*	9.79	4.32	7.21	3.15	−4.59	**<.001**
*Physical Neglect*	7.53	2.30	6.78	2.46	−1.89	.062
*Physical Abuse*	6.56	3.03	6.07	2.49	−1.22	.226
*Sexual Abuse*	6.24	2.98	5.81	2.17	−1.11	.270
Dissociative Experiences Scale	36.20	17.14	23.68	15.10	−5.97	**<.001**
*Amnesia*	24.98	18.09	14.72	15.48	−4.29	**<.001**
*Absorption*	47.49	18.29	33.44	17.84	−5.78	**<.001**
*Depersonalization/Derealization*	32.95	24.30	19.30	18.65	−4.30	**<.001**
Somatoform Dissociation Questionnaire	34.89	12.43	28.74	9.28	−3.80	**<.001**
Internet Gaming Disorder Scale—Short Form	24.41	8.73	18.41	7.25	−5.21	**<.001**

Bold values denote statistical significance. Italics denote names of subscales.

^*^Independent *t*-test.

ADHD, attention-deficit hyperactivity disorder.

**Table 2.** Pearson Product-Moments Correlation Coefficients

	1	2	3	4	5	6
1. ASRS	1.00					
2. WURS	0.58^**^	1.00				
3. CTQ	0.28^**^	0.40^**^	1.00			
4. DES	0.46^**^	0.47^**^	0.39^**^	1.00		
5. SDQ	0.34^**^	0.34^**^	0.47^**^	0.65^**^	1.00	
6. IGDS9-SF	0.40^**^	0.27^**^	0.15^**^	0.24^**^	0.18^**^	1.00

^**^ *P *< .01.

ASRS, Adult ADHD Self-Report Scale; WURS, Wender Utah Rating Scale; CTQ, Childhood Trauma Questionnaire; DES, Dissociative Experiences Scale; SDQ, Somatoform Dissociation Questionnaire; IGDS9-SF, Internet Gaming Disorder Scale–Short Form.

**Table 3.** Two Hierarchical Regression Analyses on Internet Gaming Disorder (N = 376)

***Step 1***				***Step 1***			
**Model 1**	**β**	***t***	***P***	**Model 2**	**β**	***t***	***P***
Probable ADHD	0.29	5.87	**<.001**	Probable ADHD	0.29	5.87	**<.001**
***Step 2***				***Step 2***			
Probable ADHD	0.27	5.40	**<.001**	Probable ADHD	0.27	5.19	**<.001**
CTQ	0.09	1.86	.06	CTQ EN	0.01	0.19	.85
				CTQ EA	0.05	0.69	.49
				CTQ PN	0.01	0.17	.87
				CTQ PA	−0.003	−0.05	.96
				CTQ SA	0.07	1.15	.25
***Step 3***				***Step 3***			
Probable ADHD	0.24	4.60	**<.001**	Probable ADHD	0.23	4.40	**<.001**
CTQ	0.04	0.67	.51	CTQ EN	−0.004	−0.07	.94
				CTQ EA	0.03	0.47	.64
				CTQ PN	0.005	0.07	.95
				CTQ PA	0.006	0.08	.93
				CTQ SA	0.06	1.06	.29
DES	0.15	2.28	**.023**	DES Amn	−0.19	−2.26	**.024**
				DES Abs	0.22	2.76	**.006**
				DES Dep/Der	0.08	1.02	.31
SDQ	0.01	0.14	.89	SDQ	0.02	0.26	.79

Bold values denote statistical significance. Probable ADHD: 0, absent; 1, present.

ADHD, attention-deficit hyperactivity disorder; CTQ, Childhood Trauma Questionnaire; DES, Dissociative Experiences Scale; SDQ, Somatoform Dissociation Questionnaire; CTQ EN, Emotional Neglect subscale of CTQ; CTQ EA, Emotional Abuse subscale of CTQ; CTQ PN, Physical Neglect subscale of CTQ; CTQ PA, Physical Abuse subscale of CTQ; CTQ SA, Sexual Abuse subscale of CTQ; DES Amn, Amnesia subscale of DES; DES Abs, Absorption subscale of DES; DES Dep/Der, Depersonalization/Derealization subscale of DES.
